# Reference Genome Assembly of the Big Berry Manzanita (*Arctostaphylos glauca*)

**DOI:** 10.1093/jhered/esab071

**Published:** 2021-11-24

**Authors:** Yi Huang, Merly Escalona, Glen Morrison, Mohan P A Marimuthu, Oanh Nguyen, Erin Toffelmier, H Bradley Shaffer, Amy Litt

**Affiliations:** 1 Department of Botany and Plant Science, University of California, Riverside, Riverside, CA 92521, USA; 2 Department of Biomolecular Engineering, University of California, Santa Cruz, Santa Cruz, CA 95064, USA; 3 UC Davis Genome Center, DNA Technologies and Expression Analysis Cores, University of California, Davis, CA 95691, USA; 4 Department of Ecology & Evolutionary Biology, University of California, Los Angeles, Los Angeles, CA 90095-7239, USA; 5 the La Kretz Center for California Conservation Science, Institute of the Environment and Sustainability, University of California, Los Angeles, Los Angeles, CA 90095-7239, USA

**Keywords:** California Conservation Genomics Project, California Floristic Province, CCGP, chaparral, conservation, endemic

## Abstract

*Arctostaphylos* (Ericaceae) species, commonly known as manzanitas, are an invaluable fire-adapted chaparral clade in the California Floristic Province (CFP), a world biodiversity hotspot on the west coast of North America. This diverse woody genus includes many rare and/or endangered taxa, and the genus plays essential ecological roles in native ecosystems. Despite their importance in conservation management, and the many ecological and evolutionary studies that have focused on manzanitas, virtually no research has been conducted on the genomics of any manzanita species. Here, we report the first genome assembly of a manzanita species, the widespread *Arctostaphylos glauca*. Consistent with the genomics strategy of the California Conservation Genomics project, we used Pacific Biosciences HiFi long reads and Hi-C chromatin-proximity sequencing technology to produce a *de novo* assembled genome. The assembly comprises a total of 271 scaffolds spanning 547Mb, close to the genome size estimated by flow cytometry. This assembly, with a scaffold N50 of 31Mb and BUSCO complete score of 98.2%, will be used as a reference genome for understanding the genetic diversity and the basis of adaptations of both common and rare and endangered manzanita species.

In this genome release, we report on the first assembled genome of a member of the genus *Arctostaphylos.* Our genome assembly is part of the California Conservation Genomics Project (CCGP), the goal of which is to establish patterns of genomic diversity across the state of California and its many habitats. The CCGP will sequence the complete genomes of approximately 150 carefully selected species projects. Many of these taxa are threatened or endangered, and therefore in need of conservation management in the face of rapidly accelerating biodiversity decline. The combined reference genome plus landscape genomics approach of the CCGP, based on the resequencing of many individuals of each target species across the state, will allow the identification of hotspots of diversity across California and provide a framework for informed conservation decisions and management plans.

Manzanitas (*Arctostaphylos* Adans) are among the most conspicuous and dominant native chaparral species in the California Floristic Province (CFP), a biodiversity hotspot (Howell 1957) characterized by a Mediterranean-type climate with hot, dry summers and cool, wet winters. These plants comprise the most diverse woody genus in the CFP (Minnich and Howard 1984; Baldwin et al. 2012), and their diversity has long fascinated (and perplexed) taxonomists. Manzanitas serve essential roles in their native ecosystems, including rapidly regenerating in fired-disturbed areas, and providing food resources for pollinators and fruit-eating animals (Fulton and Carpenter 1979; Minnich and Howard 1984; Kauffmann et al. 2015). In addition, these plants are of great importance for conservation management: over half of the more than 100 morphologically defined manzanita species and subspecies are narrow endemics with highly restricted distributions and are considered rare and/or endangered (Baldwin et al. 2012; Kauffmann et al. 2015; https://www.rareplants.cnps.org/).

In contrast to their importance in ecology, evolution, and conservation studies, genomic resources for manzanitas are nearly nonexistent beyond investigations into karyotypes of diploid (2*n* = 2*x* = 26) and tetraploid (2*n* = 4*x* = 48) species (Wells 1968; Kruckeberg 1977; Rosatti 1981; Schierenbeck et al. 1992; Baldwin et al. 2012). In this study, we present the first genome sequence of a manzanita. Big berry manzanita, *Arctostaphylos glauca* (Figure 1), is a widespread diploid species common in northern Baja California and across southern and coastal central California that is hypothesized to be the progenitor of several potential hybrid manzanita species (Parker 2007). With funding and support from the CCGP, we created this scaffold-level assembly using a hybrid *de novo* assembly approach that combines Hi-C chromatin-proximity and PacBio HiFi long-read sequencing data. This genome assembly will provide a robust basis for studying the diversification and evolutionary history of *Arctostaphylos* in the CFP.

**Figure 1.**
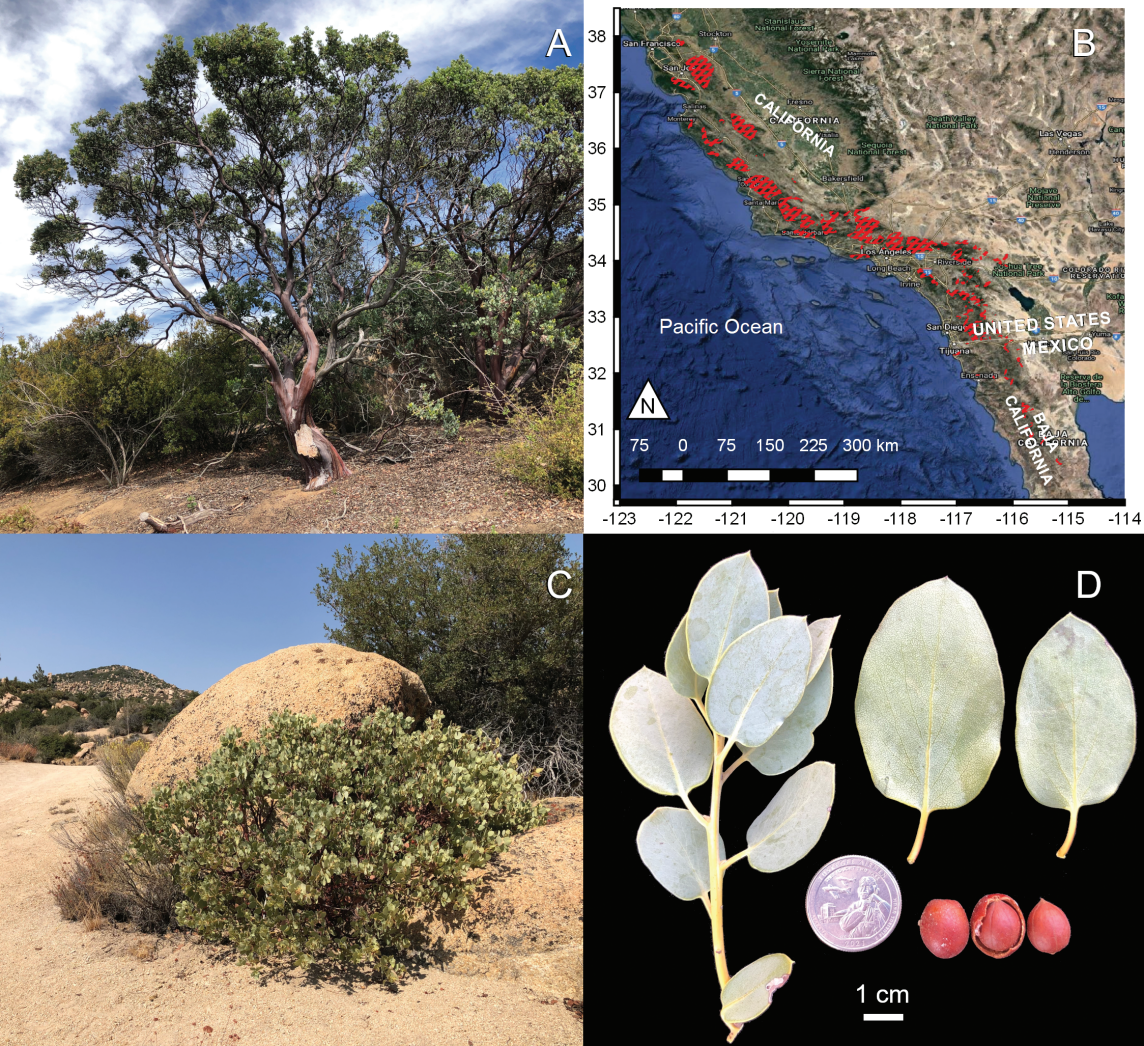
*Arctostaphylos glauca*, big berry manzanita. (A) A tall, arborescent individual in montane chaparral; (B) range map of *A. glauca*. The areas colored in red show the approximate range of *A. glauca*, based on georeferenced herbarium collections in the Consortium of California Herbaria database (CCH2.org). Satellite imagery is from Google Earth. (C) Low shrubby growth form, in a particularly dry habitat; (D) stem, leaves, and fruit. The two detached leaves show the adaxial (left) and abaxial (right) leaf surfaces. Scale bar is 1 cm long, and the coin placed for scale is a US 25 cent piece (quarter), 24.26 mm in diameter. Photograph taken on a uniform background, which was then digitally removed. Materials moved slightly in digital editing, to condense the image, while preserving scale. All photos were taken by, and are property of author GM.

## Methods

### Biological Materials

We selected for sequencing a naturally occurring big berry manzanita adult (34.20649°N, 117.78815°W) located in the San Gabriel Mountains, Angeles National Forest, Los Angeles County, California. We collected floral buds and inflorescences from this individual on February 19, 2018, and January 20, 2020. When possible, we extracted from floral tissue, which was easier to grind and gave higher yields of DNA than other tissues. Tissues were flash-frozen in liquid nitrogen and stored at −80 °C prior to DNA extraction. A voucher is deposited (UCR ACC. # 292491) at the herbarium of University of California, Riverside (UCR).

### Nucleic Acid Library Preparation and Sequencing

#### Hi-C Library

A Dovetail Hi-C library was prepared in a similar manner as previously described (Lieberman-Aiden et al. 2009). For each library, chromatin was fixed in place with formaldehyde in the nucleus. Extracted, fixed chromatin was digested with DpnII, the 5′ overhangs were filled in with biotinylated nucleotides, and free blunt ends were ligated. After ligation, crosslinks were reversed, and the DNA purified from protein. Purified DNA was treated to remove biotin that was not internal to ligated fragments. The DNA was then sheared to ~350 bp mean fragment size and sequencing libraries were generated using NEBNext Ultra enzymes and Illumina-compatible adapters. Biotin-containing fragments were isolated using streptavidin beads before PCR enrichment of each library. The libraries were prepared and sequenced on an Illumina HiSeq X by Dovetail Genomics (Santa Cruz, CA).

#### Pacific Biosciences HiFi Library

High molecular weight (HMW) genomic DNA (gDNA) was extracted from a 750 mg sample of young floral buds following the protocol described in Workman et al. (2018) with the minor modification of using the nuclear isolation buffer (NIB) supplemented with 350 mM Sorbitol (NIB-S) to resuspend the ground tissue and during the first wash of the nuclei pellet. The integrity of the HMW DNA was evaluated using the Femto Pulse system (Agilent Technologies, Santa Clara, CA). Purity of the DNA was assessed by 260/280 and 260/230 absorbance ratios on a NanoDrop spectrophotometer.

For PacBio library preparation, 11 ug of HMW gDNA were sheared to an average size distribution of ~16 kb mode using Diagenode’s Megaruptor 3 system (Diagenode, Belgium; cat. B06010001). Sheared DNA was quantified by Quantus Fluorometer QuantiFluor ONE dsDNA Dye assay (Promega, Madison, WI; cat. E6150) and the size distribution was checked by Agilent Femto Pulse (Agilent Technologies, Santa Clara, CA; cat. P-0003-0817). The sheared gDNA was concentrated using 0.45× of AMPure PB beads (Pacific Biosciences - PacBio, Menlo Park, CA; cat. 100-265-900). Concentrated, sheared gDNA was quantified by Quantus Fluorometer QuantiFluor ONE dsDNA Dye assay (Promega, cat E6150). A HiFi library was constructed using the SMRTbell Express Template Prep Kit v2.0 (PacBio, cat. 100-938-900) according to the manufacturer’s instructions. 6 ug of sheared, concentrated DNA was used as input for the removal of single-strand overhangs at 37° for 15 min, followed by further enzymatic steps of DNA damage repair at 37° for 30 minutes, end repair and A-tailing at 20° for 10 min and 65° for 30 min, ligation of overhang adapter v3 at 20° for 1 h and 65° for 10 min to inactivate the ligase, and nuclease treatment of SMRTbell library at 37° for 1 h to remove damaged or non-intact SMRTbell templates (SMRTbell Enzyme Cleanup Kit, PacBio, cat. 107-746-400). The SMRTbell library was purified and concentrated with 1X Ampure PB beads (PacBio, cat. 100-265-900) for size selection using the BluePippin system (Sage Science, Beverly, MA; cat BLU0001). The input of 2.2 ug purified SMRTbell library was used to load into the Blue Pippin 0.75% Agarose Cassette (Sage Science, cat BLF7510) using cassette definition 0.75% DF Marer S1 3–10 kb Improved Recovery for the run protocol. Fragments >7 kb were collected from the cassette elution well. The size-selected SMRTbell library was purified and concentrated with 0.8× AMPure beads (PacBio, cat 100-265-900). The 17 kb average HiFi SMRTbell library was sequenced at UC Davis DNA Technologies Core (Davis, CA) using a single 8M SMRT Cell and Sequel II sequencing chemistry 2.0 on a PacBio Sequel II sequencer.

### Genome Assembly

#### Nuclear Genome Assembly

We assembled the genome of the big berry manzanita following a protocol adapted from Rhie et al. (2021) as part of the CCGP assembly efforts. The CCGP assembly protocol version 1.0 uses PacBio HiFi reads and Hi-C chromatin capture data for the generation of high-quality and highly contiguous nuclear genome assemblies. The output corresponding to a diploid assembly consists of two pseudo haplotypes (primary and alternate). The primary assembly is more complete and consists of longer phased blocks. The alternate consists of haplotigs (contigs of clones with the same haplotype) in heterozygous regions and is not as complete and more fragmented. Given the characteristics of the latter, it cannot be considered on its own but as a complement of the primary assembly (https://lh3.github.io/2021/04/17/concepts-in-phased-assemblies, https://www.ncbi.nlm.nih.gov/grc/help/definitions/).

To generate this assembly, we removed remnant adapter sequences from the PacBio HiFi dataset using HiFiAdapterFilt [Version 1.0] (Sim 2021) and assembled the initial set of contigs with the filtered PacBio reads using HiFiasm [Version 0.13-r308] (Cheng et al. 2021) (see Table 1 for assembly pipeline and relevant software). Next, we identified sequences corresponding to haplotypic duplications and contig overlaps on the primary assembly with purge_dups [Version 1.0.1] (Guan et al. 2020) and transferred them to the alternate assembly. We aligned the Hi-C data to both primary and alternate assemblies using the Arima Genomics Mapping Pipeline (https://github.com/ArimaGenomics/mapping_pipeline) and scaffolded the genomes using SALSA [Version 2, options --e GATCGATC] (Ghurye et al. 2017; Ghurye et al. 2019). We closed the generated gaps in both assemblies using the PacBio HiFi reads and YAGCloser [commit 20e2769] (https://github.com/merlyescalona/yagcloser). The primary assembly was manually curated by iteratively generating and analyzing Hi-C contact maps. To generate the contact maps, we aligned the Hi-C data against the corresponding reference with bwa mem [Version 0.7.17-r1188, options -5SP] (Li 2013), identified ligation junctions, and generated Hi-C pairs using pairtools [Version 0.3.0] (Goloborodko et al. 2018). We generated a multi-resolution Hi-C matrix in binary form with cooler [Version 0.8.10] (Abdennur and Mirny 2020) and balanced it with hicExplorer [Version 3.6] (Ramírez et al. 2018). We used HiGlass [Version 2.1.11] (Kerpedjiev et al. 2018) and the PretextSuite (https://github.com/wtsi-hpag/PretextView; https://github.com/wtsihpag/PretextMap; https://github.com/wtsi-hpag/PretextSnapshot) to visualize the contact maps. Assemblies were then checked for contamination using the BlobToolKit Framework [Version 2.3.3] (Challis et al. 2020), and trimmed for remnants of sequence adaptors and mitochondrial contamination.

**Table 1.** Assembly pipeline and software usage. Software citations are listed in the text

Assembly	Software	Version
Filtering PacBio HiFi adapters	HiFiAdapterFilt https://github.com/sheinasim/HiFiAdapterFilt	Commit 64d1c7b
K-mer counting	Meryl	1
Estimation of genome size and heterozygosity	GenomeScope	2
*De novo* assembly (contiging)	HiFiasm	0.13-r308
Long read, genome-genome alignment	minimap2	2.16
Remove low-coverage, duplicated contigs	purge_dups	1.0.1
**Scaffolding**		
Hi-C mapping for SALSA	Arima Genomics mapping pipeline https://github.com/ArimaGenomics/mapping_pipeline	Commit 2e74ea4
Hi-C Scaffolding	SALSA	2
Gap closing	YAGCloser https://github.com/merlyescalona/yagcloser	Commit 20e2769
**Hi-C contact map generation**		
Short-read alignment	bwa	0.7.17-r1188
SAM/BAM processing	samtools	1.11
SAM/BAM filtering	pairtools	0.3.0
Pairs indexing	pairix	0.3.7
Matrix generation	Cooler	0.8.10
Matrix balancing	hicExplorer	3.6
Contact map visualization	HiGlass	2.1.11
	PretextMap	0.1.4
	PretextView	0.1.5
	PretextSnapshot	0.0.3
**Organelle assembly**		
Sequence similarity search	BLAST+	2.10
Long read alignment	Pbmm2 (https://github.com/PacificBiosciences/pbmm2)	1.4.0
Variant calling and consensus	bcftools	1.11-5-g9c15769
Extraction of sequences	seqtk	1.3-r115-dirty
Circular-aware long-read alignment	racon	1.4.19
Sequence polishing	raptor	0.20.3-171e0f1
Sequence alignment	lastz	1.04.08
Gene annotation	MitoFinder	1.4
**Genome quality assessment**		
Basic assembly metrics	QUAST	5.0.2
Assembly completeness	BUSCO	5.0.0
	Merqury	1
**Contamination screening**		
General contamination screening	BlobToolKit	2.3.3

#### Mitochondrial Genome Assembly

We identified a subset of mitochondrial reads from the PacBio HiFi dataset using BLAST+ [Version 2.10] (Camacho et al. 2009) by identifying regions of similarity between the reads and the mitochondrial (mito) database (National Library of Medicine (US), National Center for Biotechnology Information 2004). These mitochondrial reads were used as input in HiFiasm [Version 0.13-r308] to generate the mitochondrial assembly. Given the circularity of the mitochondrial genome, we carried out self-alignment of the sequence using lastz [Version 1.04.08] (Harris 2007) to manually identify and remove duplicated regions. We aligned the subset of mitochondrial reads to the assembly using raptor [Version 0.20.3-171e0f1] (https://github.com/isovic/raptor) and polished it with racon [Version 1.14.] (https://github.com/isovic/racon). We searched for matches of the resulting mitochondrial assembly sequence in the nuclear genome assembly using BLAST+ and filtered out scaffolds from the nuclear genome with a percentage of sequence identity >99% and size smaller than the mitochondrial assembly sequence. From the subset of mitochondrial reads used for the assembly, we analyzed the BLAST output and the species of the closest mitochondrial sequence available in the NCBI GenBank database, *Vaccinium macrocarpon* (Accession number: NC_023338.1). We used the mitochondrial assembly of *V. macrocarpon* as a guide for the mitochondrial gene annotation generated with MitoFinder [Version 1.4] (Allio et al. 2020).

#### Chloroplast Genome Assembly

We identified chloroplast reads from the PacBio HiFi dataset with BLAST+ using the plastids RefSeq genomes [v4.1] (O’Leary et al. 2016). From this subset, we analyzed the matches and identified the species of the closest chloroplast sequence available in the NCBI database as *Camellia taliensis* (NC_022264.1). Next, we found matches of the *C. taliensis* chloroplast genome sequence in the nuclear genome assembly with BLAST+ and filtered out scaffolds from the nuclear genome assembly with length smaller than the *C. taliensis* length, sequence identity >90%, and *e*-value <0.00001. We aligned the filtered scaffolds to the *C. taliensis* chloroplast genome with minimap2 (Li 2018) and generated a consensus sequence with bcftools (Li 2011). We manually curated the sequence using lastz. Finally, we polished the last assembly version using raptor and racon and annotated it using the web platform GeSeq (Tillich et al. 2017).

#### Genome Size Estimation, Quality Assessment and Repeat Element Identification

We generated *k*-mer counts (*k* = 21) from the PacBio HiFi reads using meryl [Version 1] (https://github.com/marbl/meryl). The generated *k*-mer database was then used in GenomeScope2.0 [Version 2.0] (Ranallo-Benavidez et al. 2020) to estimate genome features including genome size, heterozygosity, and repeat content. To obtain general contiguity metrics, we ran QUAST [Version 5.0.2] (Gurevich et al. 2013). To evaluate genome quality and completeness we used BUSCO [Version 5.0.0] (Simão et al. 2015; Seppey et al. 2019) with the embryophyta ortholog database (embryophyta_odb10) which contains 1614 genes. Assessment of base level accuracy (QV) and *k*-mer completeness was performed using the previously generated meryl database and merqury (Rhie et al. 2020). We further estimated genome assembly accuracy via BUSCO gene set frameshift analysis using the pipeline described in (Korlach et al. 2017). In addition, we used RepeatModeler (v1.0.11) and RepeatMasker (v4-0-7) to identify and classify repetitive elements in the assembled genome (Smit and Hubley 2008; Smit et al. 2015).

## Results

**Figure 2.**
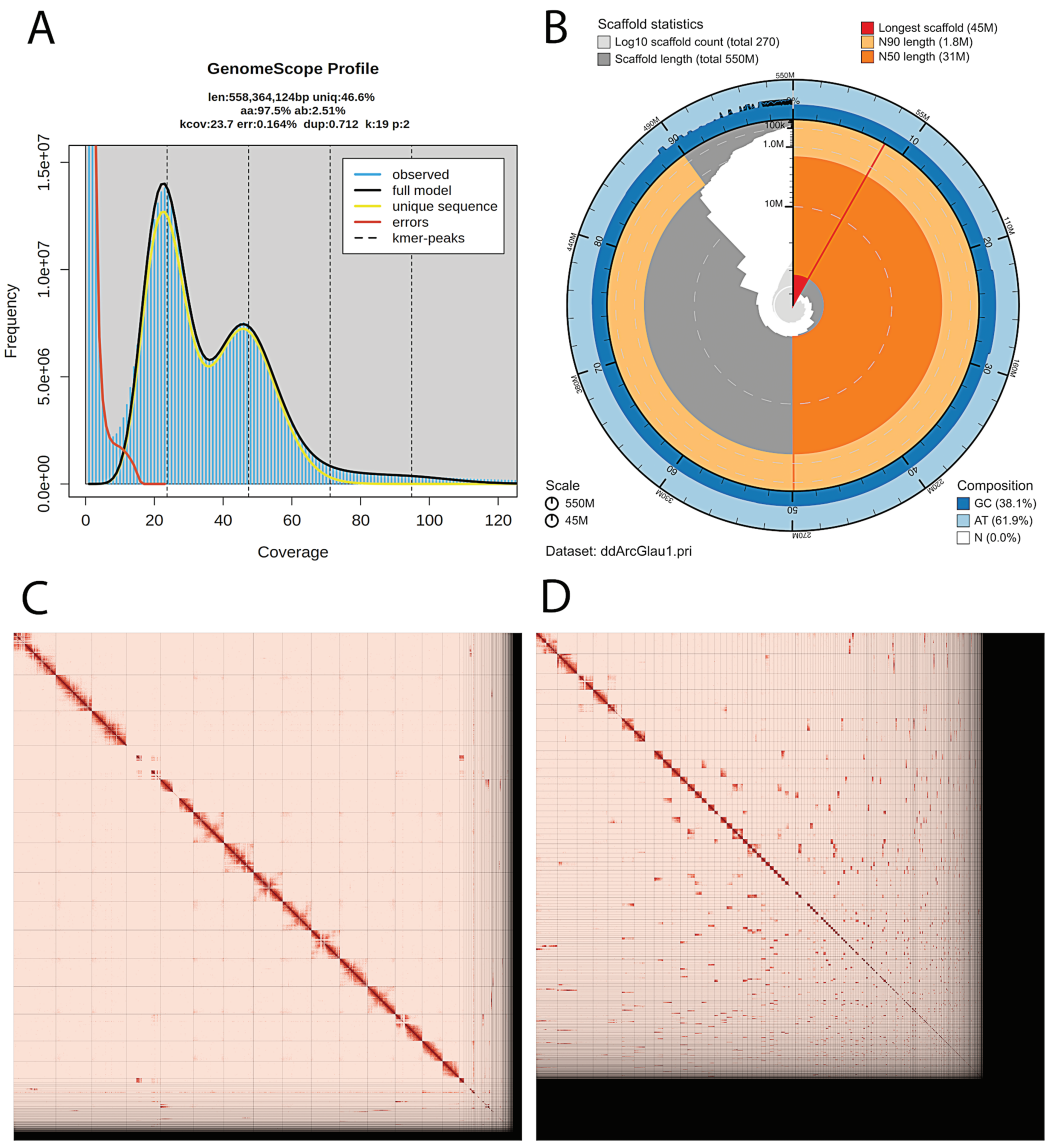
Visual overview of genome assembly metrics. (A) K-mer spectra output generated from PacBio HiFi data without adapters using GenomeScope2.0. The bimodal pattern observed corresponds to a diploid genome. K-mers covered at lower coverage but higher frequency correspond to differences between haplotypes, whereas the higher coverage but lower frequency *k*-mers correspond to the similarities between haplotypes; (B) BlobToolKit Snail plot showing a graphical representation of the quality metrics presented in Table 2 for the *A. glauca* primary assembly (ddArcGlau1). The plot circle represents the full size of the assembly. From the inside-out, the central plot covers length-related metrics. The red line represents the size of the longest scaffold; all other scaffolds are arranged in size-order moving clockwise around the plot and drawn in grey starting from the outside of the central plot. Dark and light orange arcs show the scaffold N50 and scaffold N90 values. The central light grey spiral shows the cumulative scaffold count with a white line at each order of magnitude. White regions in this area reflect the proportion of Ns in the assembly The dark vs. light blue area around it shows mean, maximum and minimum GC vs. AT content at 0.1% intervals (Challis et al. 2020); (C, D) Hi-C contact maps for the primary (C) and alternate (D) genome assembly generated with PretextSnapshot. Hi-C contact maps translate proximity of genomic regions in 3D space to contiguous linear organization. Each cell in the contact map corresponds to sequencing data supporting the linkage (or join) between two of such regions. Scaffolds are separated by black lines and higher density corresponds to higher levels of fragmentation..

We generated a *de novo* nuclear genome assembly of the big berry manzanita (ddArcGlau1) using 199 million read pairs of Hi-C data and 1.8 million PacBio HiFi reads. The latter yielded ~45-fold coverage (N50 read length 15 246 bp; minimum read length 46 bp; mean read length 14 552 bp; maximum read length of 54 291 bp). Calculation of coverage is based on a flow-cytometry estimated genome size of ~600 Mb reported in a previous study of *Arctostaphylosuva-ursi* (Siljak-Yakovlev et al. 2010). Assembly statistics are reported in tabular and graphical form in Table 2 and Figure 2, respectively.

**Table 2.** Sequencing and assembly statistics, and accession numbers

Bio projects & vouchers	CCGP NCBI BioProject		PRJNA720569			
	Genera NCBI BioProject		PRJNA721387			
	Species NCBI BioProject		PRJNA734616			
	NCBI BioSample		SAMN19489519			
	Specimen identification		​​UCR ACC. # 292491			
	NCBI Genome accessions		**Primary**		**Alternate**	
	Assembly accession		GCA_019985065.1		GCA_019985075.1	
	Genome sequences		JAHSPW000000000		JAHSPX000000000	
Genome sequence	PacBio HiFi reads	Run	1 PACBIO_SMRT (Sequel II) run: 1.8 M spots, 27.3G bases, 8.7Gb downloads			
		Accession	SRR14883332			
	Hi-C Illumina reads	Run	1 Illumina HiSeq X Ten run: 199.2M spots, 59.7G bases, 37Gb download			
		Accession	SRR14883331			
Genome assembly quality metrics	Assembly identifier (Quality code^*a*^)			ddArcGlau1 (6.7.Q62)		
	HiFi Read coverage^*b*^		45X			
			**Primary**		**Alternate**	
	Number of contigs		353		2470	
	Contig N50 (bp)		8 041 760		1 739 008	
	Longest Contigs		22 990 225		10 884 557	
	Number of scaffolds		271		2350	
	Scaffold N50 (bp)		31 280 158		3 804 428	
	Largest scaffold		45 401 621		22 987 546	
	Size of final assembly (bp)		547 548 103		556 397 040	
	Gaps per Gbp		150		1885	
	Indel QV (Frame shift)		48.36		47.39	
	Base pair QV		62.36		56.24	
			Full assembly = 58.28			
	*k*-mer completeness		74.39		65.01	
			Full assembly = 95.59			
	BUSCO completeness	**C**	**S**	**D**	**F**	**M**
	(embryophyta) *n* = 1614	98.20%	95.70%	2.50%	0.90%	0.90%
		85.90%	83.30%	2.60%	1.30%	12.80%
	Organelles		1 Partial mitochondrial sequence 1 Partial chloroplast sequence			MZ779111 XXXXXX

^*a*^Assembly quality code x.y.Q derived notation, from (Rhie et al. 2021). x = log_10_[contig NG50]; y = log_10_[scaffold NG50]; Q = Phred base accuracy QV (Quality value). BUSCO Scores. (C)omplete and (S)ingle; (C)omplete and (D)uplicated; (F)ragmented and (M)issing BUSCO genes. n, number of BUSCO genes in the set/data base. Bp: base pairs.

^*b*^Read coverage has been calculated based on a genome size of 600Mb.

The primary assembly consists of 271 scaffolds spanning 547Mb with contig N50 of 8Mb, scaffold N50 of 31Mb, largest contig of 22Mb, and largest scaffold of 44Mb. The Hi-C contact map suggests that the primary assembly is highly contiguous (Figure 2C). As expected, the alternate assembly, which consists of sequence from heterozygous regions, is less contiguous (Figure 2D). Because the primary assembly is not fully phased, we have deposited scaffolds corresponding to the alternate haplotype in addition to the primary assembly.

The final genome size (547 Mb) is close to the estimated values from the Genomescope2.0 *k*-mer spectra (estimated size of 558Mb, range 556.1–558.3Mb). The *k*-mer spectrum output shows a bimodal distribution with two major peaks, at ~24- and ~47-fold coverage, where peaks correspond to homozygous and heterozygous states respectively. This pattern corresponds to a diploid genome. Based on PacBio HiFi reads, we estimated a 0.164% sequencing error rate and 2.51% nucleotide heterozygosity rate. The assembly has a BUSCO completeness score of 98.2% using the embryophyta gene set, and a per base quality (QV) of 66. RepeatModeler indicates that the genome includes 57.71% repetitive elements. The classification of repeat elements generated by RepeatMasker is shown in Table 3.

**Table 3.** Classification of repeat elements generated from RepeatMasker

	Number of elements^*a*^	Length occupied (bp)	Percentage of sequence (%)
Retroelements	160 425	112 810 688	20.6
SINEs	17 580	3 242 835	0.59
Penelope	1155	97 740	0.02
LINEs	20 303	6 704 697	1.22
CRE/SLACS	37	1672	0
L2/CR1/Rex	4680	678 218	0.12
R1/LOA/Jockey	340	22 011	0
R2/R4/NeSL	70	3992	0
RTE/Bov-B	3670	1 765 787	0.32
L1/CIN4	9180	3 982 099	0.73
LTR elements	122 542	102 863 156	18.79
BEL/Pao	622	98 414	0.02
Ty1/Copia	49 026	41 614 407	7.6
Gypsy/DIRS1	62 124	58 039 401	10.6
Retroviral	3184	346 807	0.06
DNA transposons	288 141	58 683 503	10.72
hobo-Activator	43 650	10 654 515	1.95
Tc1-IS630-Pogo	4515	615 692	0.11
En-Spm	0	0	0
MuDR-IS905	54	53 928	0.01
PiggyBac	121	6007	0
Tourist/harbinger	4780	1 700 494	0.31
Other (mirage, P-element, P-element, Transib)	695	43 698	0.01
Rolling-circles	4313	1 802 302	0.33
Unclassified	373 015	120 296 966	21.97
Total interspersed repeats		291 791 157	53.29
Small RNA	19 997	17 205 637	3.14
Satellites	2056	628 418	0.11
Simple repeats	121 138	6 848 295	1.25
Low complexity	17 346	833 121	0.15

^*a*^Most repeats fragmented by insertions or deletions have been counted as one element.

### Mitochondrial Assembly

We generated an initial mitochondrial genome assembly with HiFiasm using a subset of HiFi reads that matched to publicly available mitochondrial reference genomes. Following an initial gene annotation with MitoFinder, we manually curated the assembly and introduced 13 gaps (*N* sequence of 100 bp) to solve partial annotation of 12 genes and re-annotated the assembly. Final mitochondrial genome size was 592 049 bp, about average for a plant mitochondrial genome. The base composition of the final assembly version is A = 27.16%, C = 22.78%, G = 22.84%, T = 26.99%, and consists of 23 transfer RNAs and 33 protein coding genes (12 of them partial). In comparison to mitochondrial genomes from other members of the Ericaceae, this assembly is similar to that of *Vaccinium macrocarpon* at 459 678 bp (Fajardo et al. 2014) but considerably smaller than that of the saprophytic *Hypopitys monotropa* (810 116 bp; Shtratnikova et al. 2020; also known as *Monotropa hypopitys*) or *Rhododendron simsii* (802 707 bp; Yang et al. 2020).

### Chloroplast Assembly

The preliminary chloroplast assembly size was 118 663 bp, which is at the beginning of the average range for a chloroplast genome (110–200 kbp; De Las Rivas et al. 2002). The base composition of the assembly is A = 31.61%, C = 18.69%, G = 18.03%, T = 31.65%, and it consists of 32 transfer RNA genes, 5 ribosomal RNA genes, and 115 protein coding genes. The chloroplast of big berry manzanita is smaller than that of *R. pulchrum* (136 249 bp; Shen et al. 2019), *R. simsii* (152 214 bp; Yang et al. 2020), *V. macrocarpon*, and four additional *Vaccinium* species (173 245 bp ~176 045 bp; Kim et al. 2020). Not surprisingly, the big berry manzanita chloroplast assembly is an order of magnitude larger than the highly reduced chloroplast genome of the saprophytic and non-photosynthesizing *H. monotropa* (35 336 bp; Gruzdev et al. 2016).

## Discussion

Our assembly is the first sequenced genome in the genus *Arctostaphylos*, representing the initial step toward understanding genetics and adaptation in this highly diversified genus. In addition to enhancing ongoing phylogenetic and conservation research, this assembly will enable investigations of the genetic basis of adaptations including drought tolerance and fire resilience. These traits, which are ecological hallmarks of manzanitas, are of growing importance in the context of the increased drought and fire frequency and intensity that are occurring in California as a result of climate change. This assembly can also serve as a reference for studying the diversification and population genetics of *Arctostaphylos*, which may shed light on aspects of diversification in other complex groups of the CFP.

*Arctostaphylos* is the third genus with a genome assembly in the heath family, Ericaceae, following release of assembled genome sequences of *Rhododendron* (two assemblies) and *Vaccinium* (one assembly) (Colle et al. 2019; Soza et al. 2019; Yang et al. 2020). These two genera are of significant economic importance: many species, hybrids, and cultivars of *Rhododendron*, including rhododendron and azalea, are important landscape and ornamental plants, and the fruits of many *Vaccinium* species, which include cranberry, blueberry, and huckleberry, are consumed by humans and other animals. Species in the Ericaceae are also notable for their ability to tolerate acidic and nutrient-poor soils that often characterize boreal forests and bogs, allowing them to thrive in habitats that are inaccessible to most species. Their tolerance for these conditions is due in part to the formation of mutualistic associations between the roots of the plants and soil fungi of a type unique to the heath family known as ericoid mycorrhizae. Ericoid mycorrhizae are distinct from common mycorrhizal associations found in most angiosperms, and are far less well understood (Watkinson 2016). Complete genome sequences from three genera in this family will provide a strong foundation for investigating the basis of this unique mutualism and its ability to promote survival in inhospitable soils.

The size of the *A. glauca* assembly is 547Mb, which is similar to the two *Rhododendron* genomes and half that of *V corymbosum*, which is tetraploid (Supplementary Table 1). The tetraploid nature of *V. corymbosum* also explains the vastly greater number of duplicated genes in its assembly compared to the two diploid assemblies. The scaffold N50 of the *A. glauca* assembly is longer than *R. williamsianum*, and close to *R. simsii* and *V. corymbosum,* suggesting that the contiguity of *Arctostaphylos* is comparable to the other taxa (Supplementary Table 1). Analysis using RepeatModeler indicated that 57.71% of the *A. glauca* genome is composed of different categories of repetitive elements (Table 3). In contrast, analysis using RepeatModeler identified only 26%, 47.5%, and 44.3% of the genome comprising repeat elements in *R. williamsianum*, *R. simsii*, and *V. corymbosum* respectively. The BUSCO completeness assessment of the *A. glauca* assembly (98%) is higher than *R. williamsianum* (89%) and close to the *V. corymbosum* (97%), indicating that our final assembly is high quality (Supplementary Table 1). Overall, the *A. glauca*, *R. simsii* and *V. corymbosum* genomes are of comparably high contiguity and completeness. The lower contiguity and completeness of the *R. williamsianum* genome may be due to the lack of HiFi or other long-read data in the assembly. This explanation is consistent with other studies demonstrating improved assembly with the inclusion of longer reads (Bradnam et al. 2013; Utturkar et al. 2014).

Although the big berry manzanita is a common and widespread species, nearly half of the 60+ manzanita species are rare or threatened. Many are now represented by only one or two populations, and are thus vulnerable to complete eradication by the increasingly common and intense wildfires experienced across California each year. Our manzanita genome sequence will help fulfill the overall goal of the CCGP, serving as a key resource to assess genetic diversity in these threatened endemics and move forward with coordinated conservation programs.

## Supplementary Material

esab071_suppl_Supplementary_Table_S1

## Data Availability

Data generated for this study are available under NCBI BioProject PRJNA720447. Raw sequencing data for sample voucher UCR ACC. # 292491 (NCBI BioSample SAMN19489519) are deposited in the NCBI Short Read Archive (SRA) under SRR14883331 for PacBio HiFi data and SRR14883332 for Hi-C Illumina short-read data. GenBank accessions for both primary and alternate assemblies are GCA_019985065.1 and GCA_019985075.1; and for genome sequences JAHSPW000000000 and JAHSPX000000000. The GenBank organelle genome assembly accession MZ779111 is for the mitochondrial genome. GitHub repository with assembly scripts is available on https://github.com/ccgproject/ccgp_assembly.
