# Supplementary material for: Reference Genome Assembly of the Big Berry Manzanita (Arctostaphylos glauca)
Source: J Hered. 2021 Nov 24;113(2):188–96. doi: 10.1093/jhered/esab071 (PMC9113465; doi:10.1093/jhered/esab071)
Supplement: esab071_suppl_Supplementary_Table_S1 [file esab071_suppl_supplementary_table_s1.docx]

Supplementary Table 1: Related species within Ericaceae for which there is a genome assembly, assembled genome sizes, N50 sizes, proportion of repetitive elements, and BUSCO statistics.

| Species | *Rhododendron williamsianum* | *Rhododendron simsii* | *Vaccinium corymbosum* | *Arctostaphylos glauca* |
| --- | --- | --- | --- | --- |
| NCBI Accession number | QEFC00000000 | WJXA00000000 | * | JAHSPW000000000 |
| Assembled genome size | 532 Mb | 529 Mb | 1.68 Gb | 547 Mb |
| Scaffold N50 | 219 Kb | 36 Mb | 36.9 Mb | 31 Mb |
| Repetitiveness | 26% | 47.5% | 44.3% | 57.71% |
| BUSCO completeness | 89% | 94% | 97% | 98% |
| Complete BUSCOs | 1,282 (89%) | 1,349 (85%) | 1,375 (95.5%) | 1,584 (98.1%) |
| Complete and single-copy BUSCOs | 1,227 (85.2%) | 1,223 (9%) | 151 (10.5%) | 1,544 (95.7%) |
| Complete and duplicated BUSCOs | 55 (3.8%) | 126 (8.8%) | 1,224 (85%) | 40 (2.5%) |
| Fragmented BUSCOs | 27 (1.90%) | 15 (1.04%) | 19 (1.30%) | 14 (0.87%) |
| Missing BUSCOs | 131 (9.10%) | 76 (5.28%) | 46 (3.20%) | 16 (0.99%) |

* There is no available NCBI accession number for the reference genome assembly of *V. corymbosum*. The final assembly of *V. corymbosum* is available on the GigaScience database GigaDB (http://dx.doi.org/10.5524/100537).
